# Cardiorespiratory Response to Six-Minute Step Test in Post COVID-19 Patients—A Cross Sectional Study

**DOI:** 10.3390/healthcare11101386

**Published:** 2023-05-11

**Authors:** Amna Omar, Arthur de Sá Ferreira, Fatma A. Hegazy, Gopala Krishna Alaparthi

**Affiliations:** 1 Department of Physiotherapy, College of Health Science, University of Sharjah, Sharjah 27272, United Arab Emirates; 2 Postgraduate Program in Rehabilitation Sciences, Augusto Motta University Center, UNISUAM, Rua Dona Isabel 94, Bonsucesso, Rio de Janeiro 21032-060, Brazil; 3 Faculty of Physical Therapy, Cairo University, Cairo 12613, Egypt

**Keywords:** COVID-19, 6 minute step test T, 6 minute walk test, cardiorespiratory responses, functional capacity

## Abstract

Background and purpose: New coronavirus disease 2019 (COVID-19) can cause persistent symptoms and physical weakness that can lead to a limitation in activities of daily living (ADL). There is a lack of evidence about the performance in the six-minute step test (6MST) of post-COVID-19 patients and healthy subjects. The aim of this study is to investigate the cardiorespiratory response induced by the 6MST in post-COVID-19 patients and compare it with the response of the six-minute walk test (6MWT). Methods: This cross-sectional study was conducted on 34 post-COVID-19 patients and 33 healthy subjects. The assessment was performed at one month from a non-severe SARS-CoV-2 infection. Both groups were assessed by using the 6MST, 6MWT, and the pulmonary function test (PFT). Post COVID functional status (PCFS) scale was used for the post-COVID-19 group to assess functional status. Physiological responses; heart rate (HR), respiratory rate (RR), oxygen saturation (SpO_2_), blood pressure (BP), and Borg scale for fatigue and dyspnea were recorded before and after the 6MST and 6MWT. Results: the performance of the post-COVID-19 group was worse than the healthy group in both tests. In 6MWT, the distance walked by the post-COVID-19 group (423 ± 7) was 94 m less than the healthy group, and the number of climbed steps in the 6MST (121 ± 4) was 34 steps less than the healthy group. Both results were statistically significant (*p* < 0.001). There was a moderate positive correlation between the 6MST and 6MWT in walked distance versus steps number (r = 0.5, *p* < 0.001). In addition, there was a moderate correlation between the two tests in the post (HR, RR, SpO_2_, systolic blood pressure SBP, diastolic blood pressure DBP, dyspnea, and fatigue) with *p* < 0.001. Conclusions: Six-minute step tests produced similar cardiorespiratory responses when compared to a 6MWT. The 6MST can be used as an assessment tool for COVID-19 patients to evaluate their functional capacity and ADL.

## 1. Introduction

Coronavirus disease (COVID-19) is an infection caused by severe acute respiratory syndrome coronavirus 2 (SARS-CoV-2) that causes respiratory illness in the majority of cases with different severity. In December 2019, the SARS-CoV-2 began to spread rapidly all over the world, causing a global pandemic as declared by World Health Organization (WHO) in March 2019. The first confirmed case in the United Arab Emirates (UAE) was announced on 29 January 2020 in an old Chinese woman [1].

COVID-19 is a multisystemic disease, whereas the respiratory system is the major involvement that is affected by the virus. The inflammatory response to COVID-19 starts in the respiratory tract, and this inflammation can spread to other systems and cause lesions to organs that can persist for a long time [2]. The severity of COVID-19 illness ranges from mild symptoms causing flu-like symptoms to severe life-threatening illness. Approximately 80% of cases are asymptomatic or mild, and about 20% of cases are severe that required hospitalization and intensive care due to acute respiratory distress syndrome (ARDS) and many cases died because of severe sepsis and multiple organ failure [3,4]. Regardless of the severity of the acute illness, abnormal lung function was reported in 2 to 12 weeks from the onset of infection such as restrictive lung or obstruction of the airways, reduced diffusion capacity and impaired gas exchange [2,5].

Persistent symptoms can develop after recovery from SARS-CoV-2 infection which is called post-COVID-19 syndrome or long COVID-19. Studies have revealed about 60% of patients can develop at least one symptom of post-COVID-19 syndrome that can last for weeks or months. The most common post-COVID-19 symptoms are fatigue, shortness of breath, cough, myalgia, and body weakness (asthenia) which can affect patient activities of daily living (ADL) [6,7].

It was well described the limitation of cardiorespiratory fitness and pulmonary function as complications of COVID-19 that led to persistent fatigue and weakness [8]. So, it has been an interesting subject for medical research to assess functional capacity and cardiorespiratory response in post-COVID-19 patients to develop a rehabilitation program to minimize the impact of COVID-19 on patients’ activities of daily living.

The functional capacity and physical performance of individuals with pulmonary disorders can be evaluated using a variety of outcome measures. The 6MWT is considered a gold-standard test that is commonly used in hospitals to assess a submaximal level of exercise capacity. It is objective, tolerable, and can reflect the activity of daily living and quality of life. It was developed by the American Thoracic Society (ATS) and the guideline was published by ATS in 2002 [9]. Evidence showed excellent validity and reliability of 6MWT in chronic obstructive pulmonary disease (COPD) patients [10,11]. However, there might be space constraints in clinics to perform 6MWT because it needs 100 feet (30 m).

The six-minute step test (6MST) is an alternative simple test that can be used to assess functional capacity without any spatial constraints. It has an excellent intra and inter-rater reproducibility as revealed in some studies on COPD [12,13]. The 6MST has a similar physiological response to 6MWT and a good correlation between them in COPD [14,15].

There is a lack of evidence about the performance in the 6MST of post-COVID-19 patients and healthy subjects. There has been no study to determine whether the 6MST can differentiate the functional capacity and cardiorespiratory response of post-COVID-19 and healthy individuals using 6MWT as a functional performance standard. So, the aim of this study is to investigate the cardiorespiratory response induced by the six-minute step test in post-COVID-19 patients and compare it with the response to the six-minute walk test.

## 2. Methodology

A cross-sectional study was conducted on the UAE population, Sharjah region, after obtaining approval from the Research Ethics Committee of the University of Sharjah [REC-21-06-15-02-S].

Eligible subjects were selected based on inclusion and exclusion criteria, and two groups were established (post-COVID-19 group and Matched-Healthy group).

### 2.1. Inclusion Criteria

- Patients with a positive polymerase chain reaction (PCR) test for COVID-19 which was confirmed by a physician.
- Past 4 weeks from the onset of acute illness (post-recovery phase).
- Mild to moderate condition of COVID-19 based on the COVID-19 severity index [16] (mild conditions score 0–2 and moderate score 3–5)
- Age (above 18) and gender-matched subjects will be recruited from the community for a healthy group.

### 2.2. Exclusion Criteria

- Subjects with a history of pulmonary or cardiac conditions such as asthma, TB, and acute myocardial infarction.
- History of musculoskeletal conditions that interfere with the ability to perform the test such as a recent fracture or severe osteoarthritis.
- History of recurrent hemoptysis.
- Obesity, smoking, cancer, and uncontrolled diabetes mellitus.
- Subjects who are unable to perform the test for any reason such as mental illness or neuromuscular disorders.
- Subjects who are on oxygen therapy.
- Subjects who use orthosis or prosthesis.
- Subjects who have exacerbating symptoms during data collection.

### 2.3. Study Procedure

COVID-19 patients without severe symptoms were referred by the physician and the control group of healthy subjects was recruited from the community. The purpose of the study was explained to the subjects, and written informed consent was signed by them before participating in the study. COVID-19 patients were evaluated one month after the infection. All subjects underwent a standardized evaluation of the pulmonary function test according to the American Thoracic Society (ATS) and the European Respiratory Society (ERS) [17,18]. A self-report questionnaire (PCFS) was used to assess the functional status of the post-COVID-19 group. The 6MWT and 6MST were performed for all subjects, separated by 30 min for rest, in addition, two trials for each test were performed and the best result was considered. The first test was selected randomly.

### 2.4. Six-Minute Walk Test

The test was performed by all subjects under the supervision of the investigator in a 30 m enclosed straight corridor according to ATS guidelines [9]. The demographic data were collected and recorded in the worksheet for each subject before starting the test. Heart rate (HR), respiratory rate (RR), oxygen saturation (SpO_2_) and Blood pressure (BP) were measured by using a pulse oximeter. The Borg scale (0–10 points) was used for fatigue and dyspnea at the beginning and immediately after the test. Participants were asked to walk as far as possible in six minutes, with standardized instructions that were provided each minute. They were allowed to stop walking and rest during the test when they got out of breath and resumed walking when they were able to. The score of the test was the longer distance that was covered by the participant. Two tests were performed for learning and better performance with a gap of 20–30 min for rest.

### 2.5. Six-Minute Step Test

The test was performed by all subjects in an indoor hospital by using a 20 cm-high bench. Participants received standardized instructions before and during the test from the investigator [19].

The demographic data were collected and recorded in the worksheet for each subject before starting the test. Heart rate (HR), respiratory rate (RR), oxygen saturation (SpO_2_) and blood pressure (BP) were measured by a pulse oximeter. The Borg scale (0–10 points) was assessed for fatigue and dyspnea at the beginning and immediately after the test. Participants were instructed to step up and down as maximum as possible in 6 min. It was allowed to stop the test at any time for subjects who got out of breath during the test. The score of the test was the total number of steps that are performed by the participant. Two tests were performed for learning and better performance with a gap of 30 min for rest.

The sample size was calculated based upon the results of a pilot study on five subjects of post-COVID-19 patients and five subjects of healthy individuals by using the following formula:

[n = (σ_1_^2^ + σ_2_^2^) (Zα + Zβ)^2^/(m_1_ − m_2_)^2^] (1)

where Zα is the value of standard normal variate corresponding to α level of significance, Zβ is the standard normal deviate for desired power, m is the average, and σ is the standard deviation.

Zα = 1.96 (Corresponding to 95% confidence interval) and Zβ = 1.282 (Corresponding to 90% power). The mean difference in the parameters for the two groups in the 6MST was (78.0) and in 6MWT was (102.3). So, the minimum sample size for a 95% confidence level and 90% power was 30 per group.

### 2.6. Statistical Analysis

Analysis was conducted using R project version 4.2.1. Statistical evidence of significance (*p*-value) is set to <0.05. Descriptive analysis was summarized as the mean and standard deviation for quantitative variables and absolute number and percentage for categorical ones. Pre-post differences for each test on heart rate (HR), respiratory rate (RR), oxygen saturation (SpO_2_), blood pressure (BP) and modified Borg scale were analyzed by using paired t-tests. Independent t-tests were also used for testing of difference of means between groups (control vs. post-COVID-19 patients). Categorical variables were cross-tabulated and analyzed using the Chi-square test. Pearson’s coefficient was used to assess the strength of bivariate correlations between outcomes from the 6MWT (total six-minute walked distance) and 6MST (total number of steps) per group. Scatter plots with regression lines were generated to graphically represent the bivariate relationship.

## 3. Results

**Figure 1:**
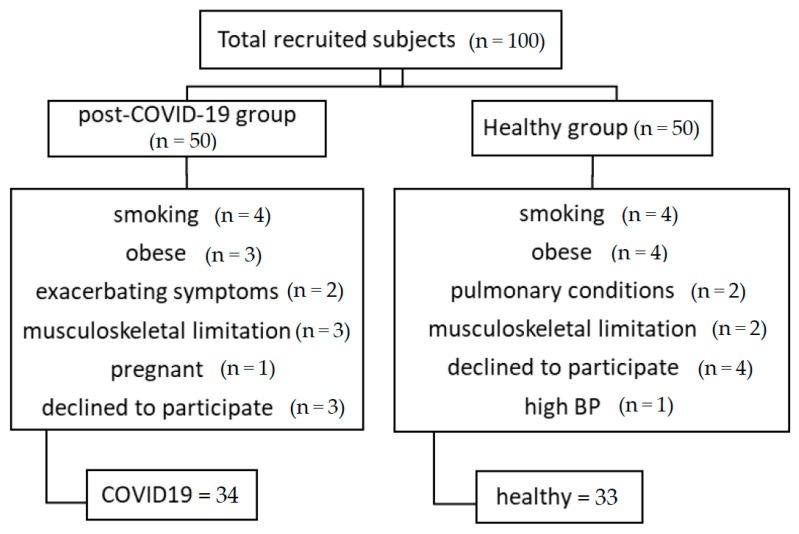
A flow chart for recruiting subjects.

In total 100 subjects were initially recruited, (post-COVID-19 = 50 and healthy = 50). Sixteen of post-COVID-19 and seventeen of the healthy subjects were excluded because of different reasons as shown in (Figure 1). A total of 67 subjects were evaluated in the study after completing all tests. Thirty-four post-COVID-19 patients were compared with thirty-three healthy matched controls. The mean age of all subjects was 35.6 ± 9 (min 20 and max 58 years).

Table 1 summarizes the demographic and baseline data of post-COVID-19 and healthy subjects. Of note, 67% were mild cases and 32% were moderate COVID-19 cases. Pulmonary functional test (PFT) results showed that there was no significant difference between healthy and post-COVID-19 groups; however, there was a slight reduction in FEV1, FVC, and PEF in post-COVID-19 patients compared to healthy.

Table 2 shows the comparison of functional capacity and post-cardiorespiratory responses between healthy and post-COVID-19 groups in the 6MWT and 6MST. The healthy group walked about 94 m more than the post-COVID-19 group in the 6MWT and 34 steps more than the post-COVID-19 group in the 6MST, both results were statistically significant (*p* < 0.001). There was a significant increase in post-(HR, RR, SBP, DBP, Borg scale for dyspnea and fatigue) in post-COVID-19 when compared to the healthy group in the 6MST and 6MWT. Post-SpO_2_ was slightly less in the post-COVID-19 group compared to the healthy in both tests; however, SpO_2_ was not statistically significant (*p* > 0.05). Additionally, post-SBP and post-DBP were non-statistically significant (*p* = 0.81, *p* = 0.24) in the 6MWT, whereas in 6MST they were significant (*p* = 0.046, *p* = 0.048).

**Figure 2:**
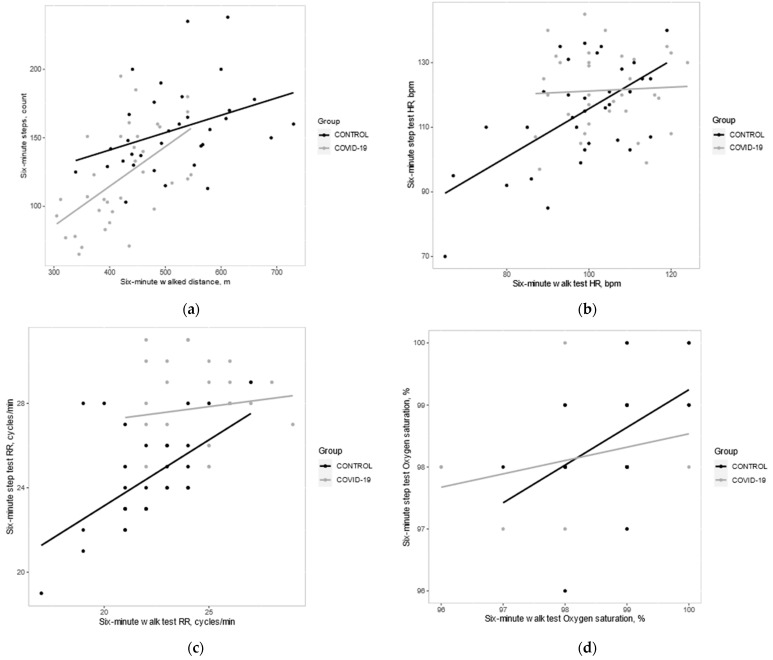

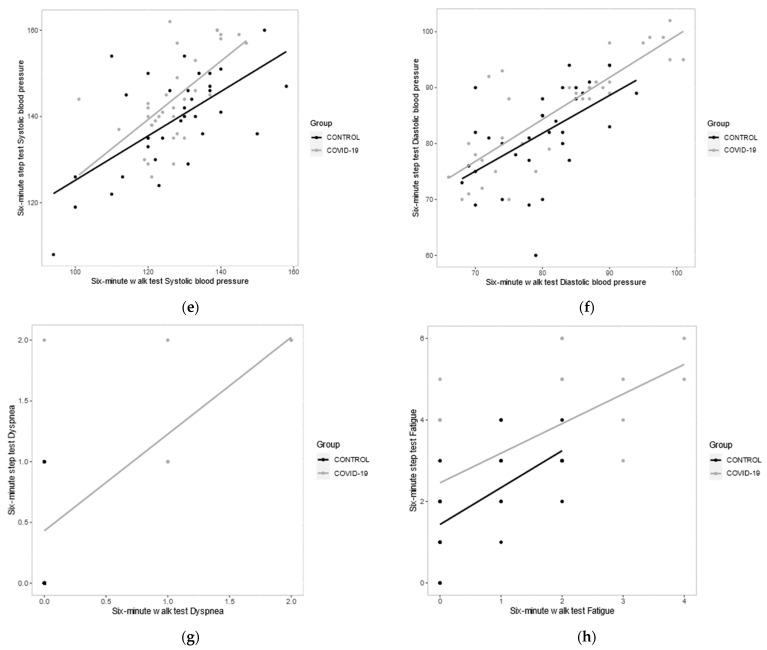
Correlation between the 6MST and 6MWT in both groups. (**a**) Distance, Steps; (**b**) Heart Rate; (**c**) Respiratory rate; (**d**) Oxygen saturation; (**e**) SBP; (**f**) DBP; (**g**) Dyspnea; (**h**) Fatigue.

The correlation between the 6MST and 6MWT was analyzed in each group and a positive relationship between the 6MST and 6MWT in all parameters (post-test) was shown in Table 3 and Figure 2. A moderate correlation was revealed between the walked distance in 6MWT and the number of climbed steps in the 6MST in the post-COVID-19 group (r = 0.55, 95%CI 0.262 to 0.750; *p* = 0.001), whereas in the healthy group, it was a weak correlation (r = 0.36, 95%CI 0.019 to 0.626; *p* = 0.39). For all secondary parameters, there was a moderate correlation between the tests in the healthy group (post-HR, RR, SpO_2_, SBP, DBP, fatigue, and dyspnea) which was statistically significant (*p* ≤ 0.001). In the post-COVID-19 group, there was a moderate to strong correlation between the two tests in post-SBP (r = 0.62, *p* = 0.001), post-DBP (r = 0.83, *p* = 0.001), post-dyspnea (r = 0.63, *p* = 0.001), and post-fatigue (r = 0.55, *p* = 0.001). On the other hand, there was a weak positive correlation between the tests in post-HR (r = 0.05, *p* = 0.78), post-RR (r = 0.13, *p* = 0.47), and post-SpO_2_ (r = 0.23, *p* = 0.194) in post-COVID-19 group.

## 4. Discussion

This study investigated if the 6MST can discriminate the functional capacity and cardiorespiratory responses in post-COVID-19 compared with the response of 6MWT. The results of the study revealed that the 6MST can differentiate between post-COVID-19 patients and healthy subjects. The average number of the steps that post-COVID-19 subjects performed was 121 ± 36 which was lesser than healthy performance with a mean difference equal to 34.6 ± 8. This result was statistically significant (*p* < 0.001). In comparison with the result of 6MWT, participants in the post-COVID-19 group also walked less than those of the healthy group with a mean difference equal to 94 ± 19 (*p* < 0.001). These findings suggest that post-COVID-19 subjects have less functional capacity when compared to healthy subjects and the 6MST was able to distinguish the group who walked a lesser distance in 6MWT. Similar results were found in two previous studies that performed the 6MST on severe COVID-19 patients. The first study was conducted by Lydia J. et al. who found decreased climbed steps with an average of 86 ± 39 steps [20] and the second study was by Bianca Setra et al. who also found a low performance of the 6MST with an average of 92.3 climbed steps [21]. However, both authors assessed severe cases of COVID-19 survivors, thus explaining the less average number of steps in their results compared to our result. The reason for the decreased level of performance in the post-COVID-19 group could be intrapulmonary or extrapulmonary causes. The main intrapulmonary causes are pulmonary dysfunction and gas exchange abnormalities. Post-COVID-19 patients develop abnormalities in pulmonary function which could be due to pathological changes in the lungs induced by the SARS-CoV-2 virus [22]. Many studies assessed pulmonary function in COVID-19 patients and found reduced diffusion capacity because of pulmonary vascular injury, and weak respiratory muscles that are associated with fatigue and low exercise capacity [23,24,25]. The extrapulmonary cause could be abnormalities in the neuromuscular system (arthralgia, myalgia, weakness) which can be occurred because of the inflammatory response that is induced by the SARS-CoV-2 virus during the acute phase of illness [26]. Bruno Ribeiro et al. identified predictive factors that impair exercise capacity after COVID-19 [27], these factors are firstly altered lung function, particularly decrease lung volume. Second is sarcopenia, low exercise capacity was associated with a reduction in muscle mass and function. Third is chronic inflammation, the study observed that patients with low exercise capacity have higher inflammatory biomarkers [28].

Physical inactivity during quarantine or medication effect can also be an external reason for low performance in the post-COVID-19 group.

The Post COVID Functional Scale (PCFS) showed 55% of post-COVID-19 subjects had a slight functional limitation affecting their ADLs as subjects reported (grades 1 and 2) in (Table 1). This result was consistent with the result of the 6MST which was a smaller number of steps compared to healthy.

Limitations during daily living activities and poor quality of life (QoL) were associated with the presence of fatigue and dyspnea in post-COVID-19 patients [29]. Fatigue and mild dyspnea were observed in this study after the test in the post-COVID-19 group (Table 3). However, there was no oxygen desaturation after the 6MWT (98.5 ± 0.9) and 6MST (98.2 ± 0.8) in the post-COVID-19 group. A similar result was found by Sperling S et al. who reported that fatigue is a common persistent symptom in mild cases of younger COVID-19 patients with normal PFT, and desaturation during 6MWT was only noted in severe post-COVID-19 cases [30].

The reasons for the presence of fatigue and dyspnea in post-COVID-19 patients could be because an autoimmune response to ACE2 may cause residual injury and abnormalities in the lungs that can lead to persistent fatigue and dyspnea [29,31].

Another reason could be psychological distress such as anxiety, insomnia, and depression which are common in COVID-19 patients. An association was found between fatigue and anxiety that was not diagnosed in participants before COVID-19 [32,33] Additionally, Hugo B et al. concluded that anxiety and depression after COVID-19 episodes increased the risk of some persistent symptoms such as dyspnea and pain [32].

It was noticed that the post-test fatigue score of the Borg scale was higher in the 6MST compared to 6MWT in both groups (Table 3). Additionally, there was a significant difference between groups in the Borg scale for dyspnea which was higher in the 6MST (mean SD = 0.62 ± 0.7, *p* < 0.001) than in 6MWT (mean SD = 0.24 ± 0.6, *p* = 0.017) as COVID-19 patients reported. It is believed stepping up and down against gravity requires more effort and higher energy expenditure than walking on a flat surface [34]. This could explain the higher score on (fatigue and dyspnea) the Borg scale that participants reported after the 6MST.

The cardiorespiratory response, post-HR and post-RR were higher in post-COVID-19 than the healthy group in both tests and were statistically significant (*p* = 0.04, *p* < 0.001; Table 3). It was hypothesized that palpitation in post-COVID-19 could be due to abnormality in the autonomic nervous system that is induced by immune response during SARS-CoV-2 infection [35].

This study revealed a moderate positive correlation between the 6MWT and 6MST in the post-COVID-19 group and the healthy group (Figure 2). This result was similar to many studies that were conducted on COPD patients [12,14,15]. As noticed in Figure 2, the correlation between the walked distance in 6MWT and the number of climbed steps in the 6MST was stronger in the post-COVID-19 group (r = 0.55, *p* = 0.001) than the healthy group (r = 0.36, *p* = 0.039). Additionally, the correlation between the tests of post-SBP, DBP, dyspnea, and fatigue was stronger in the post-COVID-19 (r = 0.63, r = 0.83, r = 0.63, r = 0.55; *p* < 0.001) compared to the healthy group. Therefore, the 6MST can be used for the evaluation of COVID-19 patients as 6MWT to assess functional capacity.

The strength of the present study is that it included mostly mild COVID-19 cases, whereas other studies recruited severe COVID-19 patients. On the other hand, this study has some limitations, firstly, the assessment of the short-term effect of COVID-19; the participants were assessed at 4 weeks after the infection. Secondly, the kind of treatment and medication that patients used during the acute phase of infection were not observed, this may influence the performance of the subjects. Thirdly, a specific questionnaire for depression and anxiety was not used in this study. Further studies are needed to investigate the long-term effect of non-severe COVID-19 on other populations. Future studies can be carried out to investigate the functional capacity of a larger sample size for all different severities of COVID-19 by using the 6MST.

## 5. Conclusions

A six-minute step test produced similar cardiorespiratory responses when compared to a six-minute walk test. The 6MST can be used as an assessment tool for COVID-19 patients to evaluate their functional capacity and physical activities of daily living.

## Figures and Tables

**Table 1:** Demographic characteristics.

Characteristics	Healthy (N = 33)	Post-COVID-19 (N = 34)	*p* Value
Gender:			0.690
Female (N%)	19 (57.6%)	16 (47.1%)	
Male (N%)	14 (42.4%)	18 (52.9%)	
Age (years)	34.8 ± 8.85	36.3 ± 9.29	0.808
Body height (cm)	166.4 ± 10.6	166.6 ± 8.4	0.993
Body weight (kg)	70.8 ± 12.5	72.9 ± 13.3	0.800
BMI (kg/m^2^)	25.5 ± 3.18	26.1 ± 3.12	0.747
COVID-19 severity:			
Mild (N%)	NA	23 (67.6%)	-
Moderate (N%)	NA	11 (32.4%)	-
PCFS (score):			
Grade 0 (N%)	NA	15 (44.1%)	-
Grade 1 (N%)	NA	12 (35.3%)	-
Grade 2 (N%)	NA	7 (20.6%)	-
Grade 3–4 (N%)	NA	0 (0%)	-
Pulmonary Functional Test:			
FEV1 (L)	3.21 ± 0.761	2.81 ± 0.655	0.080
FVC (L)	3.91 ± 0.950	3.42 ± 0.858	0.094
FEV1/FVC (%)	82.3 ± 5.72	83.5 ± 10.0	0.849
PEF (L/S)	6.58 ± 1.95	5.81 ± 1.75	0.236

NA: non-applicable. BMI: Body Mass Index PCFS: post-covid functional scale. FEV1: forced expiratory volume in the first second. FVC: forced vital capacity. FEV1/FVC: forced expiratory ratio. PEF: peak expiratory flow.

**Table 2:** Comparison of functional capacity and post-cardiorespiratory responses between healthy and post-COVID-19 group in 6MST and 6MWT.

Variables	Healthy (N = 33) (Mean ± SD)	Post-COVID-19 (N = 34) (Mean ± SD)	Mean Difference ± S.E	*p*-Value
6MST:				
Number of steps	156 ± 31.7	121 ± 35.6	34.6 ± 8.2	<0.001 *
Post-HR	114.2 ± 16.1	121.4 ± 12.1	−7.2 ± 3.5	0.042 *
Post-RR	24.4 ± 2.3	27.8 ± 1.9	−3.4 ± 0.5	<0.001 *
Post-SpO_2_	98.5 ± 0.9	98.2 ± 0.8	0.2 ± 0.2	0.239
Post-Systolic BP	139 ± 11.5	144.4 ± 10.1	−5.4 ± 2.6	0.046 *
Post-Diastolic BP	81.7 ± 8.4	86.1 ± 9.4	−4.4 ± 2.2	0.048 *
Borg scale (dyspnea)	0.061 ± 0.24	0.62 ± 0.7	−0.557 ± 0.13	<0.001 *
Borg scale (fatigue)	2.12 ± 1.2	3.44 ± 1.6	−1.32 ± 0.35	<0.001 *
6MWT:				
Distance (m)	518 ± 90	423 ± 68	94.1 ± 19.4	<0.001 *
Post-HR	98.0 ± 13.1	104 ± 10.3	−5.9 ± 2.9	0.043 *
Post-RR	22 ± 2	24.4 ± 1.9	−2.4 ± 0.5	<0.001 *
Post-SpO_2_	98.7 ± 0.8	98.5 ± 0.9	0.2 ± 0.2	0.272
Post-Systolic BP	126.7 ± 14.5	127.5 ± 9.4	−0.7 ± 3	0.805
Post-Diastolic BP	79.8 ± 6.9	82.4 ± 10.4	−2.6 ± 2.1	0.238
Borg scale (dyspnea)	0 ± 0	0.235 ± 0.55	−0.24 ± 0.1	0.017 *
Borg scale (fatigue)	0.76 ± 0.83	1.35 ± 1.2	−0.59 ± 0.25	0.022 *

6MST: six-minute step test, 6MWT: six-minute walk test, HR: Heart Rate, RR: Respiratory Rate, SpO_2_: Oxygen saturation, SBP: Systolic Blood Pressure, DBP: Diastolic Blood Pressure. * *p* < 0.05 is considered statistically significant.

**Table 3:** Correlation between 6MWT and 6MST in Functional capacity and post-cardiorespiratory results in each group.

Correlation between:	Healthy (N = 33)		Post-COVID 19 (N = 34)	
	r	*p* Value	r	*p* Value
(6MWT distance vs. 6MST steps)	r = 0.360 95% CI 0.019 to 0.626	0.039 *	r = 0.552 95% CI 0.262 to 0.750	0.001 *
Post-HR of 6MWT vs. post-HR of 6MST	r = 0.606 95% CI 0.331 to 0.786	<0.001 *	r = 0.051 95% CI −0.292 to 0.382	0.776
Post-RR of 6MWT vs. post-RR of 6MST	r = 0.549 95% CI 0.253 to 0.751	0.001 *	r = 0.129 95% CI −0.219 to 0.447	0.468
Post-SpO_2_ of 6MWT vs. post-SpO_2_ of 6MST	r = 0.539 95% CI 0.240 to 0.745	0.001 *	r = 0.228 95% CI −0.119 to 0.526	0.194
Post-SBP of 6MWT vs. post-SBP of 6MST	r = 0.647 95% CI 0.391 to 0.810	<0.001 *	r = 0.628 95% CI 0.368 to 0.797	<0.001 *
Post-DBP of 6MWT vs. post-DBP of 6MST	r = 0.558 95% CI 0.266 to 0.757	0.001 *	r = 0.832 95% CI 0.688 to 0.913	<0.001 *
Borg (dyspnea) of 6MWT vs. dyspnea of 6MST	-	-	r = 0.633 95% CI 0.375 to 0.800	<0.001 *
Borg (fatigue) of 6MWT vs. fatigue of 6MST	r = 0.63 95% CI 0.366 to 0.800	<0.001 *	r = 0.547 95% CI 0.256 to 0.747	0.001 *

* *p* < 0.05 is considered statistically significant. There is no correlation and *p*-value of Borg dyspnea in healthy group because of zero dyspnea pre and post-test with a zero-standard error of the difference. 6MST: six-minute step test, 6MWT: six-minute walk test, HR: Heart Rate, RR: Respiratory Rate, SpO_2_: Oxygen saturation, SBP: Systolic Blood Pressure, DBP: Diastolic Blood Pressure.

## Data Availability

The data that was collected and analyzed in this study are available on request from the corresponding author.
